# Notes of Two Cases of Tetanus

**Published:** 1873-03

**Authors:** B. S. Herndon

**Affiliations:** Savannah, Ga.


### NOTES OF TWO CASES OF TETANUS.

BY B. S. HERNDON, M.D., SAVANNAH, GA.

CASE I.—William Young, a farmer, aged fifty-three, of robust health, temperate habits, and regular mode of life, fell asleep under a tree, in the sitting posture, on the 8th September, 1868. He was waked by a sharp pain in a spot on his left hand, which he thought was probably caused by a burn from his pipe. Pain extended up the arm, but there was no spot of injury, and no swelling nor mark of inflammation. He was very uncomfortable that night, and suffered all the following day from pain, now extending up the arm to the trunk, neck and shoulder, but he did not lie by.

On the 10th, he found himself troubled with difficulty in swallowing, and a tremor of the left arm set in. He took a purgative, and afterward a dose of laudanum, but finding no relief, sent for his family physician.

On the 11th, Dr. Washington found him suffering greatly from spasm of the muscles of the left arm and trunk, with difficulty of breathing and inability to swallow. His thirst was great, but the effort to swallow fluids occasioned severe spasms. So great was the convulsive jerking of the left arm, that an attendant had to hold it down strongly. There was also a slight jerking of the right arm. The lower extremities were unaffected.

On the 12th, I saw him him in consultation. He was cheerful and said: "Doctors, if you will remove this difficulty about my throat, I shall be all right." There was no fever nor tetanic countenance, but the spasms were very severe, occasioning piercing cries. On the approach of a paroxysm he would cry out, "Raise me up," when he would try to dislodge some viscid mucus from the throat. He had great dread of attempting to swallow anything, and waves of air caused much apprehension and distress. His thrist was great, but he dared not attempt to allay it. He called for his pipe, and would enjoy a whiff or two when the terrible spasms would allow him. The convulsive paroxysms continued through the day, and at 5 P. M. he died by apnœa. On the day before his death he walked with his wife to a place near by, where he pointed out the spot for his burial, being assured that death was nigh.

The treatment consisted of hypodermic morphia, cups to the spine, and purgatives. Chloroform distressed him greatly, provoking spasm of the muscles of deglutition. Chloral was not given. The symptoms were so much like those of hydrophobia, that inquiry was made if he had ever received a bite from a dog, but he replied in the negative. There was no autopsy.

Dr. Foot, in an interesting case of idiopathic tetanus, reported in the September number of the *Dublin Journal of Medical Science*, states that, next to wounds, the exciting cause of tetanus is exposure to wet and cold. The death rate in India is seventy-six per cent. in idiopathic, and seventy-two in traumatic cases.

Post-mortem examination in Dr. Foot's case showed neither congestion, effusion nor thickening in the membranes of the spinal cord. The substance of the cord was of natural consistence, but the color of its white parts was in places decidedly pinker than usual. The nerve cells of the gray matter, microscopically examined, were quite normal.

Case II.—Johnson Young, colored, aged fourteen, received a blow from a brickbat thrown at him. He was struck in the forehead. The wound bled a little, but seemed trivial, and did not confine him. On Sunday, the 16th of February—ten days after the injury—he complained of stiffness of the jaws, with some difficulty of swallowing, and pain in the back of the neck, the head being drawn to one side.

On the 17th, I saw him and found trismus and opisthotonos well marked. There was some, but not much pain in the *region* of the diaphragm; *no* tetanic distortion of countenance. The lower extremities were paralyzed, their muscles not being included in the tonic or paroxysmal spasm; upper extremities also exempt; abdominal muscles somewhat affected, but not greatly; pulse ninety, skin cool, intelligence perfect. Wound covered by a scab as large as a penny piece. He was purged with calomel and hippo, and took six grains of opium in the day and night, with little effect.

18th.—Tetanic symptoms were developed; complete opisthonos, and jaws nearly closed. The paroxysms of clonic spasm were violent, occasioning piercing cries. Pulse and temperature of skin normal. Ordered twenty grain doses of hydrate of chloral pro re nata, milk punch and beef tea.

19th.—He took four doses of chloral, with marked benefit; general condition the same; continue chloral with nourishment.

20th.—Tetanus persistent; clonic spasms notably relieved by chloral, of which one hundred and sixty grains were taken in the twenty-four hours.

21st.—Clonic spasm the same, with much distress about the larynx; pulse, temperature and intelligence good; takes beef tea and milk punch kindly; clonic spasm kept in check by chloral, of which one hundred and eighty grains were taken in the day and night.

22d.—Condition unchanged. Ordered castor oil, which, not acting, was followed by a turpentine enema with good effect. Full doses of quinine and morphine were substituted for chloral.

23d.—Utter failure of quinine and morphine; chloral resumed with capital effect—one hundred and eighty grains were given.

24th.—No material change; chloral, nourishment and stimulants continued.

25th.—The same; paralysis of the lower extremities continues; entire command of *sphincters;* treatment continued.

26th.—Mitigation of symptoms; he seems better every other day; quinine added to chloral.

27th.—Improvement.

28th.—Improvement.

March 4th.—Convalescent.

---

The Late Lord Lytton.—The *Lancet*, January 25th, contains the following: "True to his loyalty to the medical art, the late Lord Lytton, in a document addressed to his legal representatives, stipulated that after death, or presumed death, his body should lie untouched three days on the bed on which he died; and thereafter medical men should examine him to ascertain that he was really dead, and, if so, to certify accordingly. The profession will see with satisfaction that his lordship will receive the honor of interment in Westminster Abbey."

